# CD147 Expressed on Memory CD4^+^ T Cells Limits Th17 Responses in Patients With Rheumatoid Arthritis

**DOI:** 10.3389/fimmu.2020.545980

**Published:** 2020-10-28

**Authors:** Jinlin Miao, Kui Zhang, Zhaohui Zheng, Rui Zhang, Minghua Lv, Na Guo, Yingming Xu, Qing Han, Zhinan Chen, Ping Zhu

**Affiliations:** ^1^Department of Clinical Immunology, PLA Specialized Research Institute of Rheumatology & Immunology, Xijing Hospital, The Fourth Military Medical University, Xi’an, China; ^2^National Translational Science Center for Molecular Medicine & Department of Cell Biology, The Fourth Military Medical University, Xi’an, China

**Keywords:** rheumatoid arthritis, inflammation, Th17 cells, CD147, STAT3

## Abstract

Rheumatoid arthritis (RA) is a common autoimmune disease in which T helper-type 17 (Th17) cells have been critically involved. CD147 is a T cell activation-associated molecule and is involved in T cell development. However, it remains unclear whether CD147 participates in Th17 responses in RA patients. In this study, we demonstrated that in both the RA and healthy controls (HC) groups, CD147 expression on CD4^+^ T cells was increased in CCR6^+^ and CD161^+^ subsets, and was associated with IL-17 production. Ligation of CD147 with its monoclonal antibody (mAb) strongly inhibited Th17 responses, and knock down of CD147 expression on CD4^+^ Tm cells specifically enhanced Th17 responses, triggered by coculture with *in vitro* activated monocytes from HC. Further functional studies showed that anti-CD147 mAb decreased the activation of AKT, mTORC1 and STAT3 signaling, which is known to enhance Th17 responses. Ligation of CD147 with its mAb on CD4^+^ Tm cells specifically reduced Th17 responses induced by *in vitro* or *in vivo* activated monocytes from RA patients. In collagen-induced arthritis model, anti-CD147 mAb treatment reduced the Th17 levels and severity of arthritis *in vivo*. These data suggest that CD147 plays a negative role in regulating human Th17 responses. Anti-CD147 mAb can limit the extraordinary proliferation of Th17 cells and may be a new therapeutic option in RA.

## Introduction

Rheumatoid arthritis (RA) is a systemic inflammatory disease characterized by the infiltration of antigen-presenting cells (APCs) and T cells into the joints, synovial hyperplasia, and systemic inflammation. Th17 cells (T helper cells that produce interleukin 17 [IL-17]) are thought to promote the development and pathogenesis of RA (1–3). Investigations into the mechanisms that control Th17 responses in humans are essential for a greater understanding of RA pathogenesis and therapeutics. In this regard, much has been accomplished in the last decade.

In humans, Th17 cells express CCR6 (chemokine receptor 6) and CD161 (cluster of differentiation 161), which are useful as markers of Th17 cells (4, 5). Depending on signals from APCs, Th17 cells can not only be generated from CD4^+^ naïve T cells, but also from CD4^+^ memory T (Tm) cells (6, 7). Evans et al. (6, 8) reported that *in vitro* activated monocytes, or *in vivo* activated monocytes from synovial fluid (SF) of RA patients, preferentially promoted Th17 responses in CD4^+^ Tm cells. Further, Th17 responses in this process depended on cell-to-cell contact (6, 8), the blockade of costimulatory and adhesion pathway, including CD80/CD86, CD54, or CD40, did not affect the Th17 responses (8). As induced by APCs, membrane IL-1α may be involved in the conversion of CD4^+^ Tm cells to Th17 cells (9). Further investigations are needed to assess whether any other cell membrane-derived signals are required for Th17 responses.

CD147 is a type I transmembrane glycoprotein that is broadly expressed on hemopoietic and nonhemopoietic cells (10), and is associated with a wide range of physiologic and pathologic functions, including lymphocyte development (11). CD147 has been linked to diverse pathological states in humans, including systemic lupus erythematosus and RA (12, 13). Interestingly, CD147 is strongly upregulated on T cells after activation (14) and has a critical role in thymocyte expansion and T-cell development (15, 16). Both antibody cross-linking and knockout mice assays indicated that CD147 may inhibit T cell receptor-mediated T cell activation and proliferation (14, 17). And CD147 is elevated on activated CD4^+^ T cells and negatively regulates Th17 cell differentiation in mice (18). Evidence suggests substantial similarities and differences between murine and human Th17 cell development. Thus, the functions of CD147-regulating signal pathways in human Th17 cells, especially in a setting of inflammation, remain to be identified. Therefore, this study investigated whether CD147 is involved in human Th17 responses, and its potential mechanism in RA. In addition, the therapeutic effects of anti-CD147 mAb were evaluated, using a mouse model of collagen-induced arthritis (CIA).

## Materials and Methods

### Patients and Healthy Controls

Peripheral blood (PB) samples were obtained from 31 patients with RA and 22 age- and gender-matched HC individuals (RA and HC groups, respectively; **Table 1**). Synovial fluid (SF) samples were collected from six patients with RA. All patients fulfilled the 1987 revised criteria of the American College of Rheumatology, and had never received disease-modifying drugs or corticosteroids. Disease activity was evaluated *via* the 28-joint disease activity score (DAS28). The study was approved by the Ethics Committee of Xijing Hospital, and all subjects provided written informed consent. The procedures were conducted in compliance with the Declaration of Helsinki.

**Table 1:** Basic characteristics of HC and RA patients*.

Characteristics	HC	RA
Patients, n	22	31
Age, y	35.5 (28.75–44.25)	39.0 (29.0–44.0)
Female gender, n (%)	16 (72.7)	24 (77.4)
Disease duration, mo	NA	7.0 (3.0–12.0)
Rheumatoid factor positive, n (%)	NA	22(71.0%)
Anti-CCP positive, n (%)	NA	23 (74.2%)
ESR, mm/h	NA	32.0 (19.0–58.0)
CRP, mg/dL	NA	1.12 (0.33–4.12)
DAS28	NA	5.01(3.87–5.48)

*Values are presented as median (interquartile range) unless indicated otherwise.

CCP, cyclic citrullinated peptide antibodies; CRP, C-reactive protein; DAS28, 28-joints disease activity score; ESR, erythrocyte sedimentation rate; NA, not applicable.

### Phenotypic Analysis

The following anti-human monoclonal antibodies were used for surface phenotype and intracellular cytokine staining: anti-CD4-fluorescein isothiocyanate (FITC); anti-CD4-phycoerythrin (PE); anti-CD161-PE; anti-CCR6-PE; anti-CD45RO-PE; anti-CD147-peridinin chlorophylla protein cyanine 5.5 dies (Percp–cy5.5); anti-interferon (IFN)-γ-FITC (all from BD Biosciences); and anti-IL-17A-allophycocyanin (APC; eBiosciences). Anti-mouse monoclonal antibodies included: anti-CD4-Percp; anti-IL-17A-APC; and anti-IFN-γ-FITC (all from BD Biosciences). Appropriately conjugated IgG antibodies were used as isotype controls. Cells were acquired on a FACSCalibur flow cytometer (BD Biosciences) and analyzed using Cell Quest software (BD Bioscience) and FlowJo 7.6.1 software (Tree Star).

### Intracellular Cytokine Staining

For detection of intracellular cytokine production, cells were stimulated with 50 ng/ml of phorbol myristate acetate (Sigma-Aldrich) plus 1 µg/ml of ionomycin (Sigma-Aldrich) in the presence of 10 µg/ml GolgiStop (BD Bioscience) for 6 h at 37°C and 5% CO_2_. After surface-staining of surface markers, cells were fixed and made permeable with BD Cytofix/Cytoperm solution and Perm/Wash solution in accordance with the manufacturer’s instructions (BD Biosciences).

### Cell Isolation

PB and SF mononuclear cells were isolated by Ficoll-Paque Plus density gradient centrifugation (Lymphocyte Separation Media; PAA Laboratories). For isolation of SF mononuclear cells, SF was incubated with 40 µg/ml of hyaluronidase (Sigma-Aldrich) for 30 min at 37°C, and then the pellet was dissolved in phosphate buffered saline and subjected to density gradient centrifugation. Purification of cell subsets was performed by magnetic cell sorting (Miltenyi Biotec) and confirmed by flow cytometry in accordance with the manufacturer’s instructions. Briefly, CD14^+^ monocytes (>94% pure) were isolated by positive selection using CD14 MicroBeads. The CD14^−^ fraction was used for CD4^+^ T cell isolation by using a negative depletion kit (>90% pure). Next, CD4^+^ Tm cells were positively selected *via* CD45RO microbeads (>92% pure). The remaining CD4^+^ naive T cells were further depleted of any remaining CD45RO^+^ cells by a second depletion round (>90% pure).

### Cell Coculture

Cells were cultured in RPMI medium 1640 (Gibco) supplemented with 1% penicillin/streptomycin, 1% glutamine, and 10% fetal calf serum (PAA Laboratories). For *in vitro* activation of monocytes, purified monocytes were pre-incubated with 100 ng/ml lipopolysaccharide (LPS, Sigma Aldrich) for 30 min at 37°C and 5% CO_2_. Then cells were washed twice with 10 ml of medium and recounted. For Th17 cell polarization, LPS-activated monocytes (1 × 10^5^) were cocultured with 5 × 10^5^ purified CD4^+^ T cells and 100 ng/ml soluble anti-CD3 mAb (R&D Systems) for 3 days in 24-well plates. For CD147 ligation experiments, purified CD4^+^ T cells were incubated with 10 µg/ml anti-CD147 mAb (HAb18, generated and identified in our lab) for 1 h, and then cells were washed and resuspended in medium. To determine the fold expansion, CD4^+^ T cells were labeled with 2 mM carboxy-fluorescein diacetate succinimidyl ester (CFSE; Invitrogen) before coculture.

### Flow Cytometry Analysis of Phosphorylated Proteins

After being cocultured for 3 days or stimulated with immobilized anti-CD3mAb (plates coated with 5 μg/ml) and 1 μg/m soluble anti-CD28mAb for the indicate time, cells were fixed with 2% paraformaldehyde. Surface staining was followed by permeabilization with 90% methanol, and intracellular staining with antibodies including anti-Stat3-PE, anti-pStat3 (Y705)-Alexa Fluor 647, anti-pAkt (T308)-PE, anti-pAkt (S473)-Alexa Fluor 647 (all from BD Bioscience), anti-pS6 (Ser235/236)-Alexa Fluor 488 and anti-p70S6K (Thr421/Ser424) (all from Cell Signaling Technology). The anti-p70S6K staining was followed by secondary staining with anti-rabbit-IgG-Alexa Fluor 488 (Invitrogen).

### Cytokine Analysis

The supernatants of cocultures were collected and stored at –80°C until cytokine testing was performed. The levels of IL-17 and IFN-γ were determined using Luminex technology in accordance with the manufacturer’s instructions (EMD Millipore). The results were analyzed using BioPlex Manager 4 (Bio-Rad Laboratories).

### Lentiviral Vector Transduction

As previously described (19), the Trans-Lentiviral pLKO System were used to produce lentiviral vectors expressing CD147-specific shRNA. Purified CD4^+^ Tm cells were activated with immobilized anti-CD3 (1 μg/ml) and anti-CD28 (5 μg/ml) in the presence of human recombinant IL-2 (20 U/ml; R&D). After 24 h, the medium was carefully removed, cells were infected with viral supernatants and polybrene (6 µg/ml; Millipore) and centrifuged at 500*g* for 2 h at 4°C. Then cells were incubated for 48 h at 37°C and 5% CO_2_. Next, transfected cells were selected by puromycin (Sigma Aldrich). After IL-2 withdrawal for 24 h, cells were collected to determine cell surface markers and coculture with monocytes.

### Induction of CIA and Anti-CD147 mAb Treatment

The Animal Experiment Administration Committee of the University approved the animal care and experimental procedures. Male C57BL/6 mice were purchased from the Fourth Military Medical University, Laboratory Animal Center. All animals were 9 to 11 weeks old. CIA induction was performed as previously described (20). Briefly, C57BL/6 mice were immunized with type II collagen (CII; Chondrex) emulsified in complete Freund’s adjuvant (CFA; Chondrex) on day 0 at the tail base. On day 21, a booster injection of CII emulsified with incomplete Freund’s adjuvant (IFA; Chondrex) was administered. The mice were treated *via* intraperitoneal injection of anti-mouse CD147 mAb or isotype control mAb (5 mg/kg, n = 4; R&D Systems). Antibody was given every other day for 10 days starting on day 21. Thickness of both hind paws were measured by digital caliper. Signs of arthritis were scored for each paw on a scale of 0 to 4, as previously described (20). The mean arthritis index is the total arthritis score in all mice of each group divided by the number of mice in the group. The mice were killed on day 31 for experimental analysis.

### Statistical Analysis

Differences between groups were determined using the nonparametric Mann-Whitney test. Paired samples were compared using a Wilcoxon matched-pairs signed rank sum test. Data analyses were performed using GraphPad Prism version 5.0 (GraphPad Software). For all tests, a two-sided *P* value less than 0.05 was considered significant.

## Results

### Association Between CD147 Levels and IL-17 Production in CD4^+^ T Cells

**Figure 1:**
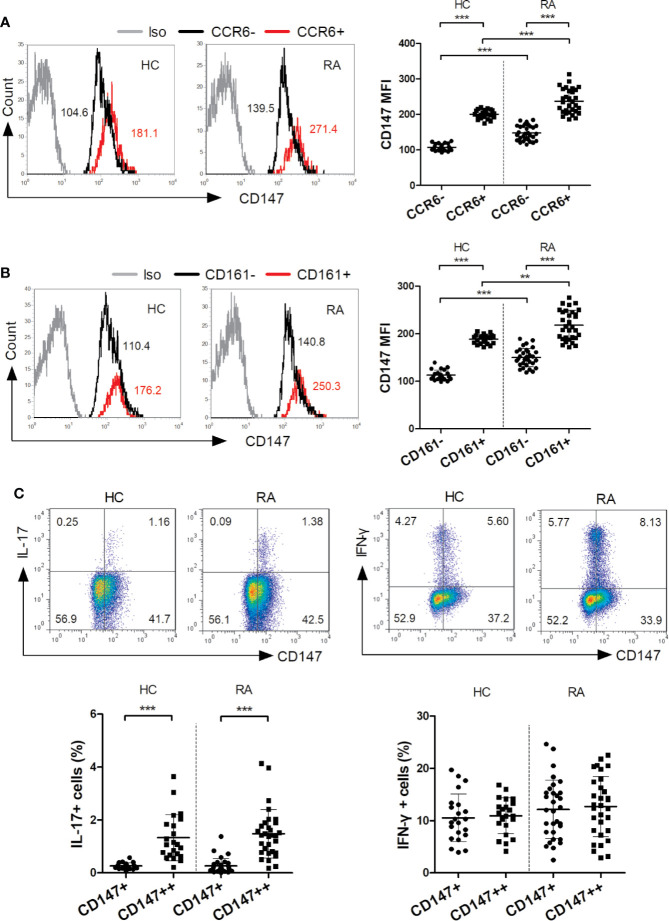
CD147 expression was associated with IL-17 production. **(A, B)** Flow cytometry was used to assess CD147 expression on circulating CD4^+^ T cell subsets, including CCR6^−/+^ cells **(A)** and CD161^−/+^ cells **(B)**, from healthy control (HC; n = 22) and rheumatoid arthritis (RA) patients (n = 31). **(C)** Flow cytometric dot-plots and cumulative data of intracellular IL-17 and IFN-γ production in CD4^+^ T cells and stratified by their CD147 expression obtained from HC (n = 22) and RA patients (n = 31). Iso, isotype control mAb; **P < 0.01; ***P < 0.001.

The main IL-17-secreting T (Th17) cells are CD4^+^CCR6^+^ and CD4^+^CD161^+^ (4, 5). In the present study, the CD147 levels of these Th17 cells were evaluated in both the RA and HC groups. In both groups but especially the RA, the CD147 levels in the CD4^+^CCR6^+^ and CD4^+^CD161^+^ cells were higher than that of the CD4^+^CCR6^–^ and CD4^+^CD161^–^ (**Figures 1A, B**). Further, in both the RA and HC groups, IL-17 production, but not IFN-γ, was mainly restricted to CD147^++^ cells (**Figure 1C**). These results supported that CD147 expression maybe associated with Th17 cells in humans.

### Effect of CD147 on Human Th17 Responses

**Figure 2:**
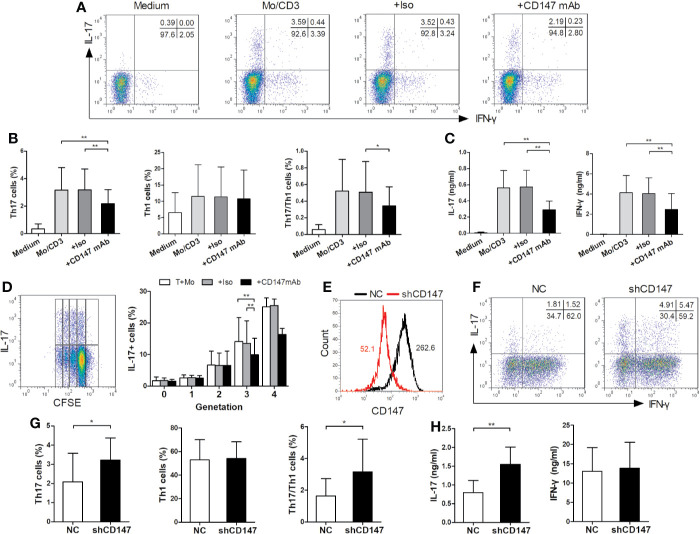
CD147 suppresses human Th17 responses. **(A–C)** CD4^+^ memory (Tm) cells from HC were cocultured with medium or LPS-activated monocytes (Mo) and anti-CD3 mAb, in absence or presence of isotype control mAb (Iso) or Anti-CD147 mAb for 3 days. Representative plots **(A)** and quantitative results of Th17, Th1 and Th17/Th1 cell percentages **(B)** in T cells, and IL-17 and IFN-γ production in cell culture supernatants **(C)** are shown (n = 9). **(D)** CFSE-labeled CD4^+^ Tm cells were cocultured with LPS-activated Mo and anti-CD3 mAb in presence of Iso or anti-CD147 mAb for 3 days and then assessed the percentage of IL-17 producing cells in each generation (generation 0–3, n = 10; generation 4, n = 4). **(E)** Representative CD147 staining histogram in CD4^+^ Tm cells from HC transduced with lentivirus expressing negative control shRNA (NC) or shRNA for CD147 (shCD147). **(F-H)** NC and shCD147 transduced CD4^+^ Tm cells were cocultured with autologous LPS-activated Mo. Representative plots **(F)** and quantitative results of intracellular expression of IL-17 and/or IFN-γ **(G)** in T cells, and IL-17 and IFN-γ levels in cell culture supernatants **(H)** were shown (n = 9). *P < 0.05; **P < 0.01.

To optimally induce Th17 response, purified bulk CD4^+^ T cells, CD4^+^ naive T cells, CD4^+^ Tm cells and autologous CD14^+^ monocytes from HC were isolated and cocultured under various conditions. The optimal condition for inducing Th17 cells is that CD4^+^ Tm cells are stimulated with LPS-activated monocytes and anti-CD3 mAb for 3 days (see online **Supplementary Figure S1**). Subsequently, CD4^+^ Tm cells, LPS-activated monocytes, and anti-CD3 mAb were cultured for 3 days, unless otherwise specified. Then to evaluate whether Th17 cells were induced from a non-committed CD4^+^ Tm cell population or reflected simply a proliferation of preexisting Th17 cells, CFSE was used to determine the proliferation of IL-17 producing cells. Indeed, the IL-17^+^ cell percentage in each generation increased with the proliferation of T cells (**Figure 2D**), indicating that LPS-activated monocytes switch non-committed Tm cells into Th17 cells.

Using the established coculture system, we investigated if CD147 ligation on CD4^+^ Tm cells could modulate Th17 responses (**Figure 2**). When compared to isotype control mAb, anti-CD147 mAb strongly decreased the percentages of Th17 and Th17/Th1 cells, whereas Th1 cell percentage was not affected (**Figures 2A, B**). In support of this, the production of IL-17 from coculture supernatants was significantly decreased by the anti-CD147 mAb (**Figure 2C**). Furthermore, the percentage of IL-17^+^ cells in the generation 0 to 2 was not affected by adding anti-CD147 mAb, but was decreased in generation 3 (**Figure 2D**). To confirm the potential role of CD147 in Th17 responses, lentiviral vectors expressing CD147-specific shRNA (LV-shCD147) were used to knock down CD147 expression in primary CD4^+^ Tm cells (**Figure 2E**). Notably, compared with the negative control shRNA (LV-NC) transduced T cells, Th17 and Th17/Th1 cell percentage and IL-17 level were noticeably higher in LV-shCD147 transduced T cells, while Th1 cell and IFN-γ levels were not affected (**Figures 2F–H**). These data collectively suggest that CD147 plays a negative role in Th17 responses.

### Effect of CD147 on AKT, mTORC1, and STAT3 Signaling

**Figure 3:**
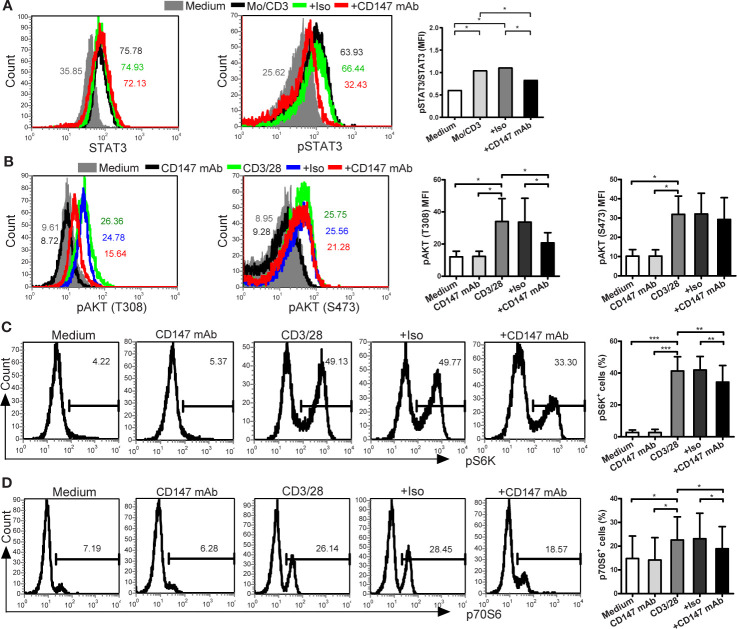
Anti-CD147 mAb inhibits AKT, mTOR and STAT3 signaling. **(A)** CD4^+^ Tm cells from HC were cocultured with medium or LPS-activated Mo and anti-CD3 mAb in presence of isotype control mAb (Iso) or anti-CD147 mAb for 3 days, then the expression of STAT3 and pSTAT3 were assessed by flow cytometry (n = 7). **(B)** Phospho-flow analysis of pAKT (T308) and pAKT (S473) in CD4^+^ Tm cells from HC with medium or anti-CD147 mAb, with or without Iso or anti-CD147 mAb stimulated with anti-CD3 and anti-CD28 mAb for 5 min (n = 7). **(C, D)** Phospho-flow analysis of pS6K (n = 7; **(C)** and p70S6 (n = 6; **(D)** in CD4^+^ Tm cells from HC with medium or anti-CD147 mAb, with or without Iso or anti-CD147 mAb stimulated with anti-CD3 and anti-CD28 mAb for 30 min. *P < 0.05; **P < 0.01; ***P < 0.001.

To evaluate the underlying mechanisms, we investigated the effect of CD147 on STAT3 (signal transducer and activator of transcription 3), a crucial regulator of Th17 lineage (21). Indeed, anti-CD147 mAb decreased STAT3 phosphorylation in CD4^+^ Tm cells that induced by coculture with LPS-activated monocytes (**Figure 3A**). It has been reported that the signaling of PI3K, Akt, and mammalian target of rapamycin (mTOR) complexes positively regulates Th17 cell development (22). Further signal analysis showed that the levels of pAKT (T308), pS6K, and p70S6, but not of pAKT (S473), induced by CD3/CD28 ligation were partly inhibited in the presence of anti-CD147 mAb in comparison to isotype control mAb (**Figures 3B–D**). This suggests that CD147 may suppress Th17 responses by affecting AKT/mTOR signaling.

### Effect of CD147 on Th17 Responses in RA Patients

**Figure 4:**
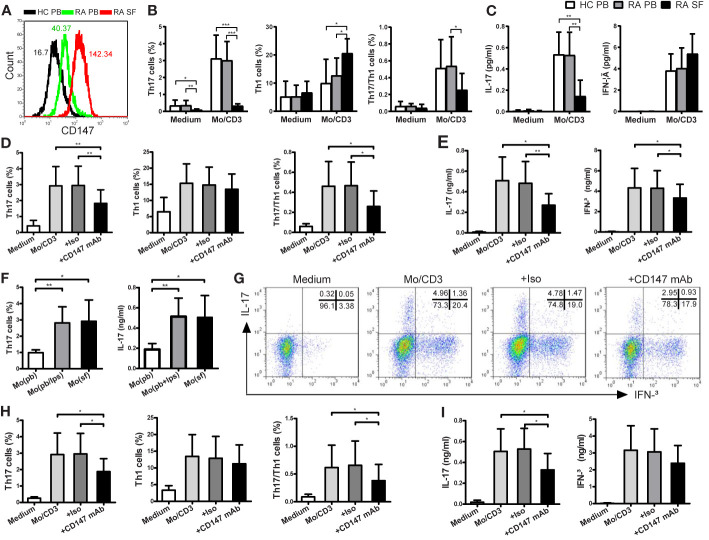
CD147 suppresses Th17 responses in patients with RA. **(A)** Representative CD147 staining histogram in CD4^+^ Tm cells from the peripheral blood of HC (HC PB), PB of RA (RA PB) and synovial fluid of RA (RA SF). **(B**, **C)** CD4^+^ Tm cells from HC PB (n = 13), RA PB (n = 12) or RA SF (n = 6) were isolated and cocultured with medium or autologous PB LPS-activated Mo and anti-CD3 mAb. **(D**, **E)** CD4^+^ Tm cells from RA PB were cocultured with medium or LPS-activated Mo in absence or presence of Iso or anti-CD147 mAb. Percentages of Th17, Th1 and Th17/Th1 cells **(B, D)** and levels of IL-17 and IFN-γ **(C, E)** are shown. **(F)** CD4^+^ Tm cells from RA PB were cocultured with autologous Mo from PB (Mo (pb)), LPS-activated Mo from PB (Mo (pb/lps)) or Mo from SF (Mo (sf)), then Th17 cell percentage and IL-17 level are shown (n = 6). **(G–I)** CD4^+^ Tm cells from RA PB were cocultured with medium or Mo from SF in absence or presence of Iso or anti-CD147 mAb. Representative plots **(G)** and quantitative results of intracellular expression of IL-17 and/or IFN-γ **(H)** in T cells, and IL-17 and IFN-γ levels in cell culture supernatants **(I)** were shown (n = 6). *P < 0.05; **P < 0.01; ***P < 0.001.

We sought to assess whether the Th17 responses triggered by monocytes were suppressed by CD147 in patients with RA. First, CD147 expression on CD4^+^ Tm cells from RA PB was higher than from HC PB, and its expression was further increased in RA SF (**Figure 4A**). Using CD4^+^ Tm cells from HC PB, RA PB and RA SF, the magnitude of monocyte-driven Th17 and Th17/Th1 responses and IL-17 production were similarly in RA PB and HC PB, but strikingly decreased in RA SF (**Figures 4B, C**). In addition, similar to the data of HC, the increase in Th17, Th17/Th1 cells and IL-17 production in RA patients triggered by LPS-activated monocytes could be suppressed by adding anti-CD147 mAb (**Figures 4D, E**). There are numerous reports that CD14^+^ monocytes from SF of RA patients were highly activated (23) and the activation status of the APCs played a crucial role in Th17 induction (6–8). To place these findings in a pathophysiological condition, we cocultured RA PB-derived CD4^+^ Tm cells with autologous PB-derived monocytes, LPS-activated PB monocytes or SF-derived monocytes. As compared to PB-derived monocytes, SF-derived monocytes increased Th17 responses to a level comparable with that of LPS-activated PB monocytes (**Figure 4F**). Furthermore, anti-CD147 mAb also significantly decreased Th17 cell percentage and IL-17 production, but not Th1 cell percentage and IFN-γ production, that triggered by SF-derived monocytes (**Figures 4G–I**).

### Anti-CD147mAb Treatment in RA Model

**Figure 5:**
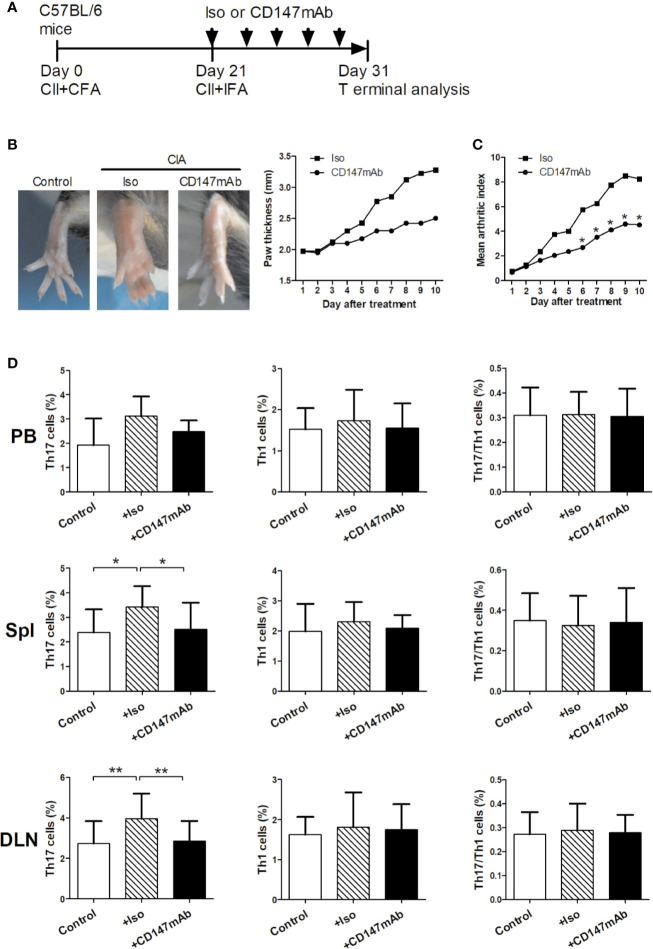
Therapeutic effects of anti-CD147mAb in CIA model. **(A)** Experimental scheme for the analysis of CIA model. C57BL/6 mice were immunized on days 0 and 21, and treated with anti-mouse CD147 mAb (CD147 mAb) or isotype control mAb (Iso) at every other day for 10 days starting on day 21. **(B)** Representative hind paws from normal mice (Control), Iso and CD147 mAb treatment CIA mice (Day 31). The average thickness of both hind paws was measured (n = 4). **(C)** The mean arthritis index in CIA mice that were administrated Iso or CD147 mAb (n = 4). **(D)** Percentages of Th17, Th1 and Th17/Th1 cell in the PB, spleens (Spl) and draining lymph nodes (DLN) from Control and CIA mice treated with Iso or CD147 mAb (n = 4). *P < 0.05; **P < 0.01.

The *in vivo* effects of anti-mouse CD147mAb were studied using a mouse model of CIA (**Figure 5**). After first immunization, on day 21, mice were treated with anti-mouse CD147mAb or isotype control mAb every other day for 10 days (**Figure 5A**). In contrast to the isotype control mAb treatment, anti-CD147mAb treatment suppressed the increase in paw swelling (**Figure 5B**). The mean arthritis index of CIA mice was significantly reduced after 7 days of anti-CD147mAb treatment (**Figure 5C**). To observe the effect of anti-CD147mAb on Th17 responses, the peripheral blood, spleen and draining lymph node were collected and analyzed after anti-CD147mAb treatment. As shown in **Figure 5D**, the CIA mice expressed higher percentages of Th17 cells in the spleen and draining lymph nodes when compared with the normal mouse. Furthermore, Th17 cell levels in the spleen and draining lymph nodes were significantly decreased in anti-CD147mAb–treated CIA mice as compared to isotype mAb group (**Figure 5D**), whereas Th1 and Th17/Th1 cell levels were not significantly changed.

## Discussion

Numerous studies have suggested that Th17 cells and IL-17 play crucial roles in the chronic inflammatory response and subsequent tissue damage in RA (1–3). Indeed, targeting Th17 and IL-17 has been shown to ameliorate chronic inflammation in mouse RA models (24). In humans, anti-IL-17 therapy improved RA signs and symptoms (25). However, there are only limited data about what drives human Th17 responses *in vivo*, particularly in human arthritic diseases. In this study, we provide evidence that CD147 expression was increased in IL-17–producing CD4^+^ T cells, which in turn acts as a negative limitation loop to suppress human Th17 responses and inhibit the generation of Th17 cells in RA.

Firstly, we investigated the association between CD147 expression and Th17 cells in humans. In both HC and RA patients, CD147 expression on CD4^+^ T cells was increased in CCR6^+^ and CD161^+^ fraction. Further, when CD4^+^ T cells were split into two groups depending on their CD147 expression, IL-17 production was mainly restricted to CD147^++^ subset in both the HC and RA patients. These phenotypic data suggest that CD147 expression on CD4^+^ T cells closely correlated with Th17 cells in HC and RA groups. Therefore, similar to a study regarding mice (18), CD147 may suppress the extraordinary expansion of Th17 cells in human.

As previously reported (26), bulk CD4^+^ T cells were cocultured with monocytes to induce Th17 responses. Our findings showed that Th17 cells were primarily induced by cocultured CD4^+^ Tm cells, but not CD4^+^ naive T cells, with activated monocytes. As previously reported (8), activated monocytes relied on cell-contact with CD4^+^ Tm cells to induce Th17 responses by performing transwell experiments (see online **Supplementary Figure S2**), suggesting membrane-derived signals are required in this process. Then we found that ligation of CD147 with its mAb in T cells impaired the Th17 responses induced from non-Th17-committed human Tm cells. Knocking down CD147 expression on CD4^+^ Tm cells enhanced Th17 responses, but did not affect the Th1 responses. Therefore, these findings showed that anti-CD147 mAb is a stimulating Ab in this work, and CD147 probably plays a negative role in regulating human Th17 responses, but not Th1 responses.

The molecular mechanisms of Th17 responses have been intensively investigated, and numerous intracellular signaling pathways and transcriptional factors have been identified, including PI3K, Akt, mTOR, and STAT3. STAT3 plays a direct and crucial role in Th17 cell specification (27). Recent work has indicated that CD147 expressed in CD4^+^ T cells inhibits Th17 cell differentiation by suppressing the IL-6/STAT3 pathway in mice (18). In humans, we demonstrated that ligation of CD147 with its mAb suppressed the STAT3 activation in CD4^+^ Tm cells induced by coculture with LPS-activated monocytes. Further, mTOR plays an important role in STAT3 activation (28) and mTOR complex 1 (mTORC1) as a central regulator of Th17 lineage commitment by coordinating metabolic and transcriptional programs (22, 29, 30). There are mTOR complexes, including mTORC1 and mTORC2, working downstream and upstream of Akt (22). mTORC1 is activated by pAKT (T308) in a PI3K-dependent manner, whereas phosphorylation of AKT (S473) is mediated by the kinase mTORC2. Here, pS6K and p70S6 were used to assess mTORC1 and pAKT (S473) was used to assess mTORC2. Our data showed that ligation of CD147 with its mAb impairs the phosphorylation of pAKT (T308), pS6K, and p70S6, but not pAKT (S473). As previously reported, although CD147 may initiate proximal signaling molecules (such as LCK and CDK1) in T cells, the exact signaling pathway of CD147 is not fully clear in human (31). And the proximal signaling of CD147 in Th17 responses are still remain to be elucidated. What is more, augmenting the results reported in mice (18), our findings highlight the importance of metabolic activity in T cell fate decision, and indicate that the importance of the CD147-AKT-mTORC1-STAT3 pathway in the negative regulation of human Th17 responses.

Although Th17 cells play important roles in the pathogenesis of RA, Th17 responses are complex and incompletely understood in RA, particularly at the sites of inflammation. In this study, we examined the CD147 expression profile and monocyte-driven Th17 responses in patients with RA. Indeed, CD147 expression on CD4^+^ Tm cells from RA SF was significantly higher than from HC PB and RA PB, which might partly explain why the monocyte-driven Th17 and Th17/Th1 responses were strongly decreased in CD4^+^ Tm cells from RA SF. However, the monocyte-driven Th1 responses were significantly higher in RA SF than in RA PB and HC PB. These results are consistent with previous report (32), wherein Th17 cell percentage was decreased in the joints of patients with RA, but Th1 cells were more abundant in the joints. In agreement with the results of HC, anti-CD147 mAb inhibited the Th17 responses and IL-17 production induced by LPS-activated monocytes in patients with RA.

It was reported that *in vitro* LPS-activated monocytes exhibit a phenotype similar to that of *in vivo*-activated SF monocytes (1–3). As previous research reported (8), we found that *in vivo*-activated monocytes, from the local sites of inflammation, spontaneously induced Th17 responses in RA patients. Further, ligation of CD147 with its mAb specifically decreased *in vivo*-activated monocytes induced Th17 responses and IL-17 production, suggesting that CD147 is involved in the switch of Tm cells into Th17 cells at the site of inflammation in patients with RA. From another perspective, previous literatures reported by our and other teams suggested that CD147 has many ligands in tumor cells, erythrocytes or immunocytes, such as cyclophilin proteins, rhoptry-associated protein 2, integrins, and CD147 (33–35). However, the ligand of CD147 for inhibit Th17 responses in RA patients are still needed further investigations to explore. Consistent with our previous report (36), in the *in vivo* work, systemic administration of anti-CD147 mAb alleviated the severity of arthritis in CIA mice. Importantly, anti-CD147 mAb treatment simultaneously decreased the Th17 levels, but not Th1 and Th17/Th1, in the spleen and draining lymph node of CIA mice, supporting that anti-CD147mAb may improve CIA symptoms by specifically inhibiting Th17 responses. Therefore, it is reasonable to infer that in addition to Th17-cell depletion or IL-17 neutralization therapy, pharmacologic ligation of CD147 may be crucial for limiting the generation of pathogenic Th17 cells, and thus to attenuate the inflammatory response in patients with RA.

In summary, our data highlight the importance of CD147 in CD4^+^ Tm cells and monocytes interactions in the shaping of Th17 responses. By suppressing STAT3 signaling, CD147 acts as a negative receptor on CD4^+^ Tm cells to inhibit human Th17 responses. Ligation of CD147 with mAb may be a potential therapeutic approach in Th17-mediated diseases.

## Data Availability Statement

The raw data supporting the conclusions of this article will be made available by the authors, without undue reservation.

## Ethics Statement

The studies involving human participants were reviewed and approved by Ethics Committee of Xijing Hospital. The patients/participants provided their written informed consent to participate in this study. The animal study was reviewed and approved by The Animal Experiment Administration Committee of the University.

## Author Contributions

JM, KZ, ZZ, and RZ performed most of the experiments and wrote the manuscript. PZ and ZC conceived the study and revised manuscript. ML and NG participated in the experiments. YX and QH were in charge of patients’ recruitment and clinical data collection. All authors contributed to the article and approved the submitted version.

## Funding

This work was supported by the National Basic Research Program of China (No. 2015CB553704) and National Natural Science Foundation of China (No. 81801599).

## Conflict of Interest

The authors declare that the research was conducted in the absence of any commercial or financial relationships that could be construed as a potential conflict of interest.
