# Supplementary material for: CD147 Expressed on Memory CD4+ T Cells Limits Th17 Responses in Patients With Rheumatoid Arthritis
Source: Front Immunol. 2020 Oct 28;11:545980. doi: 10.3389/fimmu.2020.545980 (PMC7655988; doi:10.3389/fimmu.2020.545980)
Supplement: Supplementary file 1 [file DataSheet_1.doc]

**
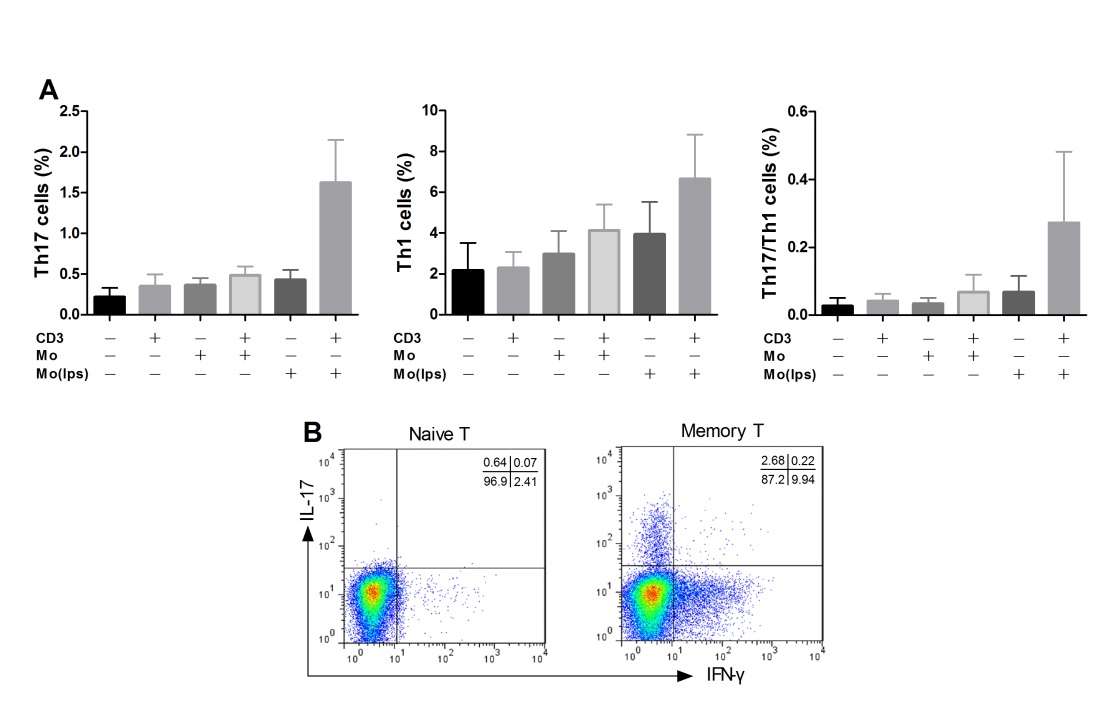
**

**Supplementary figure 1. Optimal Th17 induction in human CD4^+^ T cells. (A)** Bulk CD4^+^ T cells were cultured for 3 days with or without anti-CD3 mAb, monocytes (Mo), or LPS-activated monocytes (Mo(lps)), and examined for IL-17 and/or IFN-γ expression in CD4^+^ T cells (Th17, Th1 and Th17/Th1) (n=6). **(B)** CD4^+^ naïve and memory T cells were cocultured with autologous LPS-activated monocytes and anti-CD3 mAb for 3 days. Percent IL-17 and/or IFN-γ producing T cells are shown from one representative healthy control (n=5).


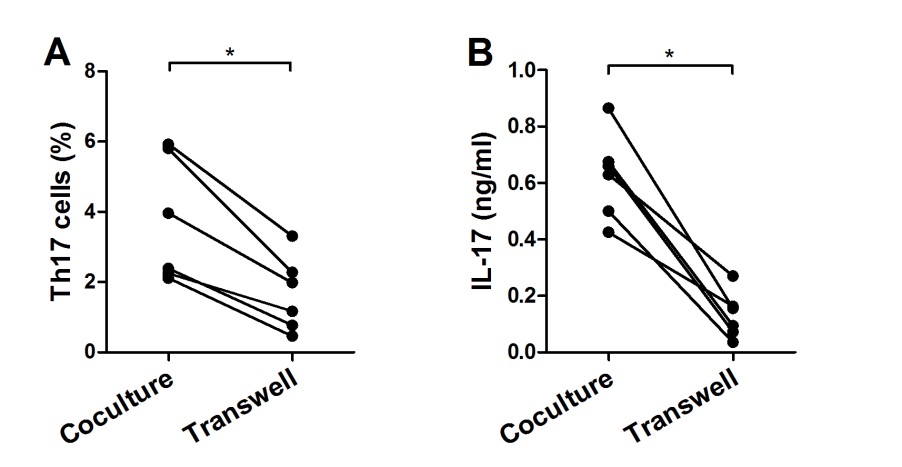


**Supplementary figure 2. The induction of Th17 responses in CD4^+^ memory T cells requires cell contact with activated monocytes. (A and B)** CD4^+^ memory T cells from peripheral blood of healthy controls (HC, n=6) were cultured with anti-CD3 mAb either in a coculture with autologous LPS-activated monocytes or in a transwell culture with a membrane separating the T cells and LPS-activated monocytes. After 3 days, the percentage of Th17 cells (A) and levels of IL-17 in cell culture supernatants (B) were shown (n=6). *, P<0.05.
